# Gallbladder Intramucosal Carcinoma Arising in a Cholesterol Polyp: A Case Report

**DOI:** 10.7759/cureus.22898

**Published:** 2022-03-06

**Authors:** Takahide Sasaki, Masatoshi Kajiwara, Fuminori Ishii, Yoshihiro Hamada, Suguru Hasegawa

**Affiliations:** 1 Gastroenterological Surgery, Faculty of Medicine, Fukuoka University, Fukuoka, JPN; 2 Pathology, Faculty of Medicine, Fukuoka University, Fukuoka, JPN

**Keywords:** gallbladder polyp, cholecystectomy, cd68, gallbladder carcinoma, cholesterol polyp

## Abstract

Cholesterol polyp is the most common benign disease of gallbladder polyps, and is considered not to be the origin of malignancy. Herein, we report a rare case of a well-differentiated adenocarcinoma arising in a gallbladder cholesterol polyp. A pedunculated mulberry-like gallbladder polyp diagnosed with a cholesterol polyp preoperatively consisted of two distinct components macroscopically: a yellow-whitish lobulated lesion and a brownish irregular lesion. Microscopically, the former revealed to be a cholesterol polyp, but the latter demonstrated a well-differentiated adenocarcinoma. Even if imaging findings suggest a gallbladder cholesterol polyp, it is important to keep in mind that carcinoma can coexist like our case.

## Introduction

Gallbladder polyps are classified into cholesterol polyp, inflammatory polyp, hyperplastic polyp, pyloric gland adenoma, intracholecystic papillary neoplasm, carcinoma and so on [1,2]. Among them, cholesterol polyp, characterized by the yellow, pedunculated and lobulated lesion, is the most common (60-90%) and categorized into the non-neoplastic benign disease [1]. Inflammatory polyp and hyperplastic polyp are also benign conditions and need no further treatment. Pyloric gland adenoma, intracholecystic papillary neoplasm and carcinoma require surgical removal via cholecystectomy and they consist of only 5% of gallbladder polyps [3]. However, it is not easy to diagnose them correctly with preoperative imaging evaluation. Clinical practice guidelines of Japan and Europe recommended that gallbladder polyp more than 10 mm in size should be removed surgically, because it is difficult to deny malignant lesions [4,5].

Herein, we report a case of gallbladder intramucosal adenocarcinoma arising in a cholesterol polyp, which was diagnosed with a cholesterol polyp preoperatively.

## Case presentation

**Figure 1:**
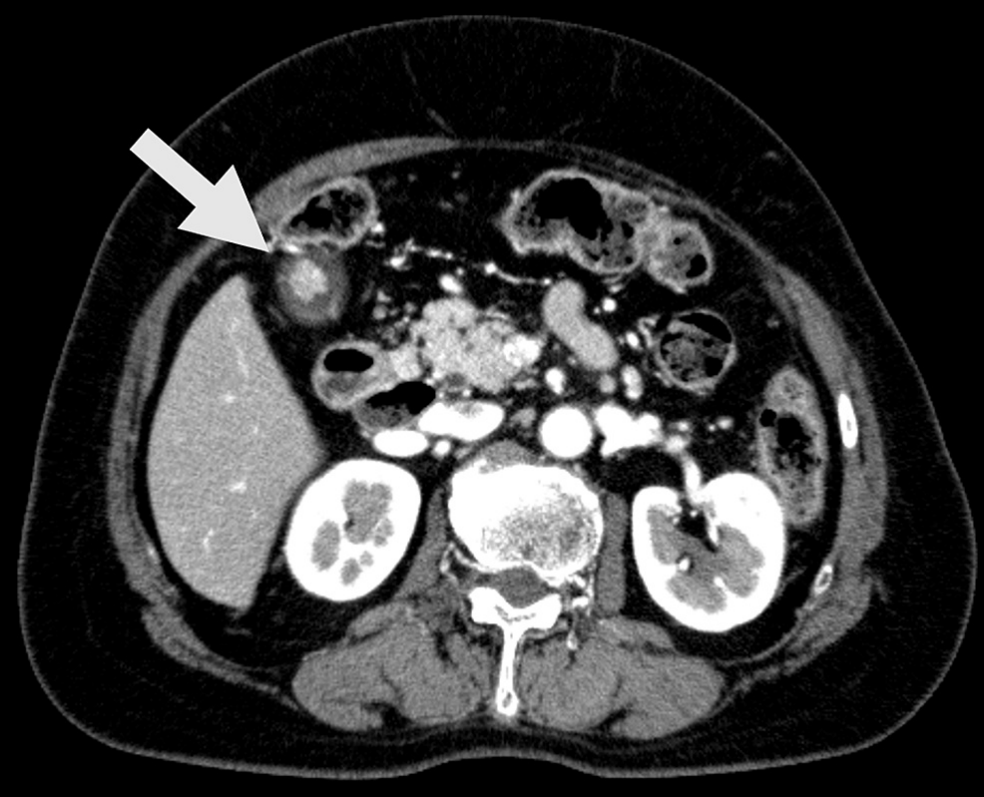
Abdominal contrast-enhanced computed tomography The arterial phase of abdominal contrast-enhanced computed tomography revealed a lobulated polyp 10 mm in size in the fundus of the gallbladder as a hyper-enhanced lesion.

A 67-year-old woman was referred to our hospital for the further evaluation of a gallbladder polyp, which was incidentally identified on abdominal ultrasonography of medical checkup at a local clinic. At the time of the visit, she did not complain of any symptoms. Laboratory data including tumor markers (CEA and CA19-9) were within normal ranges. The arterial phase of abdominal contrast-enhanced computed tomography revealed a lobulated polyp 10 mm in size in the fundus of the gallbladder as a hyper-enhanced lesion (Figure 1).

**Figure 2:**
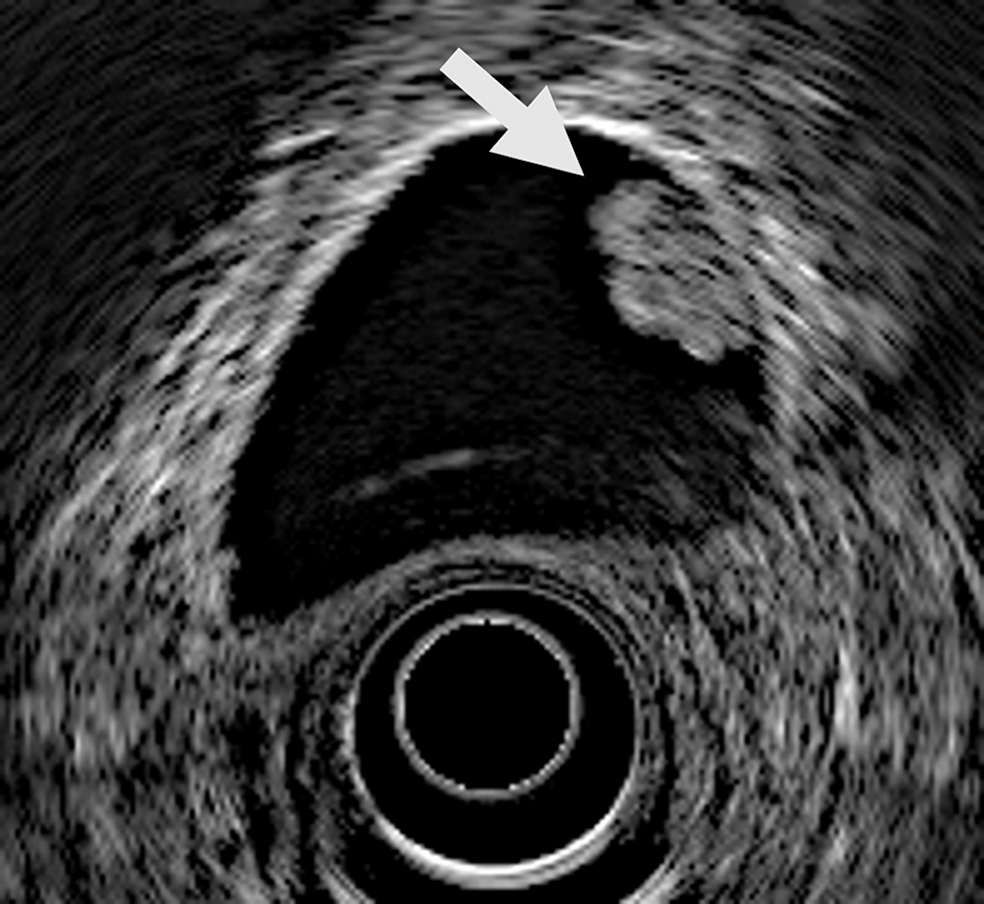
Endoscopic ultrasonography Endoscopic ultrasonography demonstrated a 10-mm brightly echogenic, pedunculated, intraluminal polypoid lesion without foci.

Endoscopic ultrasonography (EUS) also demonstrated a 10-mm brightly echogenic, pedunculated, intraluminal polypoid lesion without foci (Figure 2).

**Figure 3:**
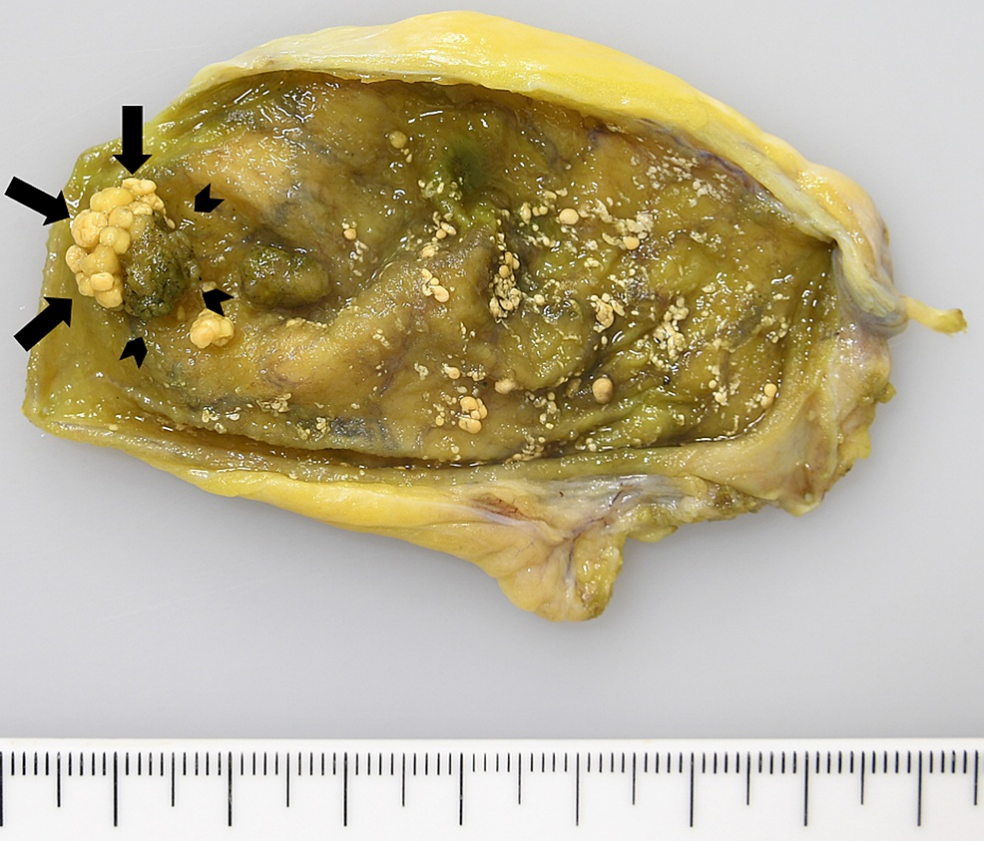
Macroscopic appearance of the resected specimens The resected specimens showed a pedunculated polyp 10 mm in size in the fundus of the gallbladder, which consisted of two distinct components; a main yellow-whitish lobulated lesion (arrows), and a brownish irregular lesion located on the proximal side of the polyp (arrowheads). Multiple small white polyps less than 5 mm were also observed from the neck to the fundus of the gallbladder.

No gallstones were detected by EUS. Color Doppler mode of EUS did not show blood flow signals inside the polyp. In addition, multiple small polypoid lesions less than 5 mm were also observed in the gallbladder. These findings were suggestive of cholesterol polyps. However, a malignant polyp could not be excluded because of its largest size of 10 mm, and laparoscopic cholecystectomy was performed following the clinical practice guidelines. The resected specimens showed a pedunculated polyp 10 mm in size in the fundus of the gallbladder, which consisted of two distinct components; a main yellow-whitish lobulated lesion, and a brownish irregular lesion located on the proximal side of the polyp. Multiple small white polyps less than 5 mm were also observed from the neck to the fundus of the gallbladder (Figure 3).

**Figure 4:**
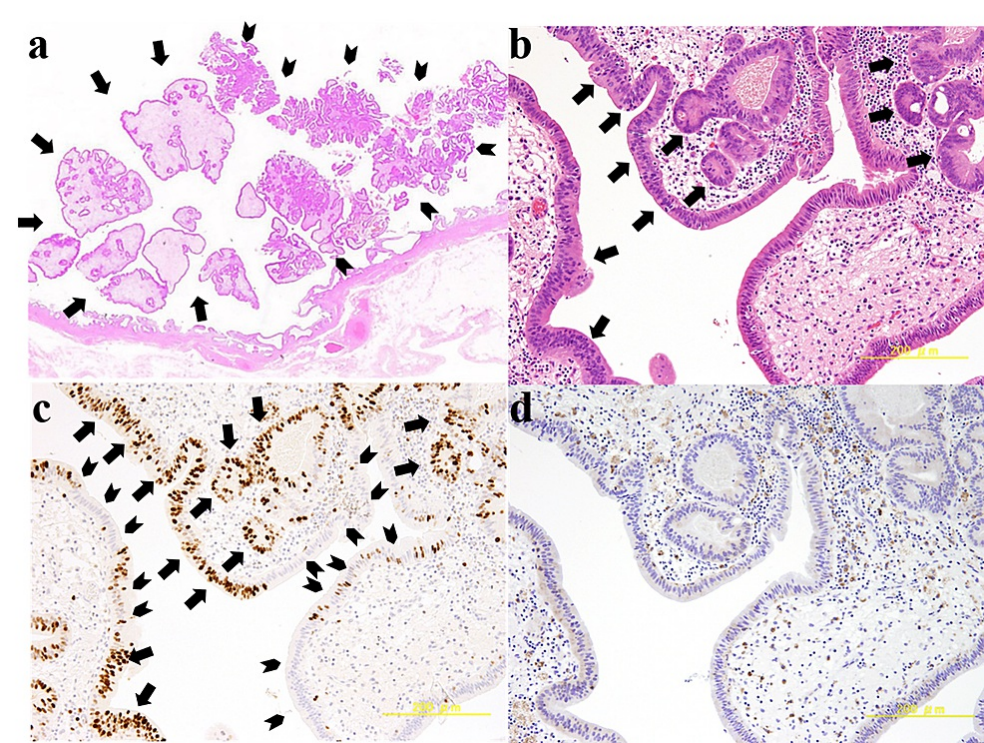
Microscopic appearance of the resected specimens a: A yellow-whitish lesion of the polyp was a cholesterol polyp, accompanying scattered foamy cells in the lamina propria (arrows). A brownish lesion of the polyp was well-differentiated adenocarcinoma (arrowheads). There were no atypical epithelial cells around the stalk of the polyp (hematoxylin and eosin, ×5). b: Transition between carcinoma and non-carcinoma parts of the polyp. Atypical epithelial cells forming irregular glandular structures were observed in carcinoma part (arrows) (hematoxylin and eosin, ×100). c: Immunological staining with a monoclonal antibody against Ki-67. Diffuse Ki-67-positive cells were identified only in the carcinoma part (arrows). Ki-67 negative cells were identified in the non-carcinoma part (arrowheads). d: Immunological staining using a monoclonal antibody against CD68. CD68-positive cells were observed in the lamina propria of both the carcinoma and non-carcinoma part.

Microscopically, CD68-positive foamy cells were scattered in the lamina propria of the yellow-whitish lesion of the polyp, which was covered by a normal epithelium. These findings are typical for the cholesterol polyp. On the other hand, brownish lesion of the polyp demonstrated proliferating atypical epithelial cells forming irregular glandular structure, and was diagnosed with well-differentiated adenocarcinoma, which was limited to the mucosal layer. Diffuse Ki-67 positive cells were identified only in the carcinoma part. Sparse CD68 positive foamy cells were also observed in the lamina propria of the carcinoma component. There were no atypical epithelial cells around the stalk of the polyp (Figure 4).

The postoperative course was uneventful and she was discharged on the third postoperative day. There is no sign of recurrence during two-year follow-up.

## Discussion

We presented a case of well-differentiated adenocarcinoma arising in a gallbladder cholesterol polyp.

European Society for Medical Oncology (ESMO) clinical practice guidelines address that gallbladder polyps ≧20 mm in size should be managed as carcinoma. Ultrasound surveillance is recommended for polyps measuring 6-9 mm (every six months for one year, then annually for five years), with resection only in enlarging polyps to 10-20 mm in size [4].

Japanese Society of Hepato-Biliary-Pancreatic Surgery (JSHBPS) clinical practice guidelines mention that prophylactic cholecystectomy should be performed for polypoid lesions of the gallbladder that are sessile, have diameters equal to or greater than 10 mm, and/or grow rapidly, considering the possibility of malignant diseases [5].

In our case, multiple pedunculated polyps with mulberry-like features were observed in the gallbladder suggesting benign cholesterol polyps. However, we could not rule out the possibility of malignant polyps because the size of the largest one was 10 mm. Then, we performed laparoscopic cholecystectomy according to the treatment guidelines. However, it is reported that 32.1% of neoplastic gallbladder polyps are smaller than 10 mm in size, and the 10 mm surgical threshold has moderate diagnostic accuracy and is insufficient to indicate surgery for neoplastic gallbladder polyps in the Dutch nationwide cohort survey [6]. We have to decide surgical indication for gallbladder polyps by not only the size but many other aspects such as singularity, wall thickening and presence of gallstones [6]. Recently, Wennmacker et al. [7] suggested the new diagnostic flowchart for gallbladder polyps based on differences in signal intensity on diffusion-weighted imaging (DWI) and apparent diffusion coefficient (ADC) value of magnetic resonance imaging (MRI) in addition to the conventional ultrasonography.

Cholesterol polyp of the gallbladder is a benign condition, but there have been some case reports about the coexistence of cholesterol polyp and adenocarcinoma or adenoma [8,9]. Akiyama et al. [8] reported a case of an 81-year-old man who was diagnosed with cholecystolithiasis and performed cholecystectomy. Carcinoma in situ was demonstrated in some parts of cholesterol polyps in the neck of the gallbladder. They discussed that the tumor epithelium might absorb cholesterol from the bile, and foamy cells were produced and deposited inside the tumor. Shimada et al. [9] presented a case of a 37-year-old man who received cholecystectomy for the pedunculated gallbladder polyp 17 mm in size. The resected specimens appeared to be typical cholesterol polyp, macroscopically. However, microscopic examinations revealed that poorly differentiated adenocarcinoma occupied approximately 60% of the stroma of the polyp, and the remaining 40% of the stroma consisted of a cluster of foamy cells. They hypothesized that cholesterol itself stimulated the mucosa of the polyp to transform into carcinoma. Although it is not a neoplastic lesion, Abukhiran et al. [10] reported a rare cholesterol polyp with osseous metaplasia. As a few cases of gallbladder carcinoma associated with cholesterol polyp had been reported so far, there is no consensus on the relationship between cholesterol polyp and carcinoma. Foamy cells are macrophages that absorbed cholesterol from the bile, and are observed in lamina propria of cholesterolosis and cholesterol polyp. In our case, foamy cells were observed throughout the lamina propria of the whole polyp. Moreover, no carcinoma cells were seen around the stalk of the polyp. These findings suggested that a cholesterol polyp was developed at first, followed by the malignant transition of the mucosal epithelium covering the polyp rather than the collision of two independent tumors; cholesterol polyp and carcinoma. If cholesterol polyp itself could be the origin of gallbladder carcinoma, a lot of gallbladder carcinomas accompanied by cholesterol polyp should have been reported. So far, some reports investigated the detailed clinicopathological correlation between gallbladder carcinoma and gallbladder polyps in the large cohort [6,11,12], whereas there was no specific description of the coexistence of cholesterol polyp and gallbladder carcinoma. Thus, the best explanation for our case appears to be that the gallbladder carcinoma had incidentally developed in the epithelium of a preexisting cholesterol polyp.

## Conclusions

As cholesterol polyps generally do not require surgical resection, differential diagnosis of polypoid lesions of the gallbladder is quite important. However, our case indicates that gallbladder cancer may coexist even if the preoperative imaging studies strongly suggest cholesterol polyp. Additional cases are necessary to evaluate the relationship between cholesterol polyps and carcinomas.
